# 

**Published:** 1917-07

**Authors:** 


### NOTE.

WE deeply regret that the full obituary notes of Mr. Walter J. Hill, M.R.C.S., L.R.C.P., who died at Clevedon on June 2nd, reached us after this issue was in the press, and will therefore be deferred until our next number.

---

James D. Staple has been appointed temporary Honorary Medical Officer to the Lyme Regis Cottage Hospital.

### Bristol Medico-Chirurgical Journal

*Established in 1883 as a Journal of the Medical Sciences for the West of England and South Wales.*

Published Quarterly (Demy 8vo, price 1/6), under the auspices of the Bristol Medico-Chirurgical Society.

### Terms for Advertisements.

| | £ | s. | d. |
|---|---|---|---|
| Whole Page ... ... ... ... | 1 | 1 | 0 |
| Half Page ... ... ... ... ... | 0 | 12 | 6 |
| Quarter Page ... ... ... ... | 0 | 7 | 6 |

Insets (2 or 4 pages), 20/-.

Discount of 5 per cent. will be allowed if payment is made at the time of ordering.

Terms for special positions upon application.

*Orders to be sent to the Publishers—*

J. W. ARROWSMITH LTD., Quay Street, Bristol.

